# A novel feature selection algorithm for identifying hub genes in lung cancer

**DOI:** 10.1038/s41598-023-48953-1

**Published:** 2023-12-07

**Authors:** Tehnan I. A. Mohamed, Absalom E. Ezugwu, Jean Vincent Fonou-Dombeu, Mohanad Mohammed, Japie Greeff, Murtada K. Elbashir

**Affiliations:** 1 School of Mathematics, Statistics, and Computer Science, University of KwaZulu-Natal, KwaZulu-Natal, King Edward Avenue, Pietermaritzburg Campus, Pietermaritzburg, 3201 South Africa; 2 Unit for Data Science and Computing, North-West University, Potchefstroom, South Africa; 3 Department of Computer Science, Faculty of Mathematical and Computer Sciences, University of Gezira, Wad Madani, 11123 Sudan; 4 School of Computer Science and Information Systems, Faculty of Natural and Agricultural Sciences, North-West University, Vanderbijlpark, South Africa; 5 Department of Information Systems, College of Computer and Information Sciences, Jouf University, 72388 Sakaka, Saudi Arabia

**Keywords:** Computer science, Information technology

## Abstract

Lung cancer, a life-threatening disease primarily affecting lung tissue, remains a significant contributor to mortality in both developed and developing nations. Accurate biomarker identification is imperative for effective cancer diagnosis and therapeutic strategies. This study introduces the Voting-Based Enhanced Binary Ebola Optimization Search Algorithm (VBEOSA), an innovative ensemble-based approach combining binary optimization and the Ebola optimization search algorithm. VBEOSA harnesses the collective power of the state-of-the-art classification models through soft voting. Moreover, our research applies VBEOSA to an extensive lung cancer gene expression dataset obtained from TCGA, following essential preprocessing steps including outlier detection and removal, data normalization, and filtration. VBEOSA aids in feature selection, leading to the discovery of key hub genes closely associated with lung cancer, validated through comprehensive protein–protein interaction analysis. Notably, our investigation reveals ten significant hub genes—ADRB2, ACTB, ARRB2, GNGT2, ADRB1, ACTG1, ACACA, ATP5A1, ADCY9, and ADRA1B—each demonstrating substantial involvement in the domain of lung cancer. Furthermore, our pathway analysis sheds light on the prominence of strategic pathways such as salivary secretion and the calcium signaling pathway, providing invaluable insights into the intricate molecular mechanisms underpinning lung cancer. We also utilize the weighted gene co-expression network analysis (WGCNA) method to identify gene modules exhibiting strong correlations with clinical attributes associated with lung cancer. Our findings underscore the efficacy of VBEOSA in feature selection and offer profound insights into the multifaceted molecular landscape of lung cancer. Finally, we are confident that this research has the potential to improve diagnostic capabilities and further enrich our understanding of the disease, thus setting the stage for future advancements in the clinical management of lung cancer. The VBEOSA source codes is publicly available at https://github.com/TEHNAN/VBEOSA-A-Novel-Feature-Selection-Algorithm-for-Identifying-hub-Genes-in-Lung-Cancer.

## Introduction

Lung cancer begins in the lung tissues and can potentially metastasize to various parts of the body^1^. It is broadly categorized into two types: small cell lung cancer (SCLC) and non-small cell lung cancer (NSCLC). The SCLC is an aggressive type of lung cancer that spreads rapidly. However, the NSCLC is considered as the most frequently diagnosed form of lung cancer, representing approximately 85% of all cases. It is known to grow and metastasize at a slower rate than SCLC^2–4^. The most prevalent risk factor for developing lung cancer is smoking, but exposure to other environmental hazards, including asbestos, radon and air pollution can also contribute to an elevated risk. Common symptoms associated with lung cancer are persistent chest pain, cough, shortness of breath, inexplicable weight loss, and fatigue^5^. Lung cancer is responsible for approximately 350 deaths per day, which is nearly 2.5 times higher than the number of deaths caused by colorectal cancer (CRC), the second leading cause of cancer deaths.

In 2023, cigarette smoking directly contributes to approximately 103,000 out of 127,070 lung cancer deaths (81%), with an additional 3560 deaths caused by second-hand smoke. If classified separately, the remaining balance of approximately 20,500 deaths not caused by smoking would rank as the eighth leading cause of cancer deaths among both genders^6^. The early identification of cancer significantly increases the chances of survival. Accurate determination of the specific type of cancer is crucial for administering appropriate treatment to the patient. Conventional techniques that involve examining various biopsy samples under a microscope are both time-consuming and not cost-effective in advanced cases, and there is a risk of obtaining false negative outcomes^7^. Observable physical traits of cells and tissues, including their size, shape, and arrangement, are known as morphological characteristics.

The traditional method of classifying cancer relied primarily on these characteristics. However, multiple studies have revealed the significant limitations of this approach. For example, similar morphological characteristics among some cancer types make it challenging to differentiate between them. Moreover, interpreting these characteristics is subjective, and there is a risk of experts' bias in tumor identification. These drawbacks can result in misdiagnosis and inadequate treatment outcomes for patients. Consequently, researchers have sought alternative methods such as gene expression data obtained from microarrays to overcome these limitations. This approach provides a more objective and comprehensive understanding of cancer at the molecular level and has shown significant potential in enhancing cancer classification and treatment^8,9^.

The mechanism through which the genetic information contained in a gene is utilized to generate a functional product is known as gene expression. This is indicative of the biochemical processes within tissues, cells, other organism’s genetic characteristics and can, therefore, play a fundamental role in the early detection of cancer. Ribonucleic acid (RNA)-sequencing and deoxyribonucleic acid (DNA) microarrays technologies that allow for measuring the expression levels of genes in a sample and produce valuable and high dimensional data for computational analysis^10^. However, gene expression data present several challenges for analysis; these include noise and high dimensionality. The number of features or genes can significantly exceed the number of samples (typically contains thousands to tens of thousands of genes), leading to a potential lack of statistical power. Class imbalance is also a common issue because this can negatively affect the performance of classification models. Moreover, only a small subset of genes may be informative for a particular disease, rendering the majority of genes irrelevant^11^.

Reducing data dimensionality is an effective solution for handling gene expression data. Feature selection techniques are commonly used to tackle this problem by selecting a minimal set of features that effectively represent the entire feature space while preserving essential information from the data. This approach reduces model training time while potentially improving classification accuracy^12,13^. There are different types of feature selection techniques. The first category is the wrapper approach, which assesses the worth of features by measuring the model’s performance using a machine learning technique. The second category, known as the filter approach, assesses the statistical properties and relevance of features without using a machine learning classifier. It avoids the necessity of training a machine learning model and cross-validation steps required by wrapper-based methods. The filter approach includes techniques like meta-heuristic algorithms, recursive feature elimination, and sequential feature selection. However, compared to the wrapper method, the filter approach is generally more efficient but may exhibit lower accuracy. The third category of feature selection methods is the embedded method, which integrates feature selection directly into the learning process. This approach treats feature selection as an inherent component and includes techniques such as decision tree-based methods and L1 regularization as notable examples^14,15^. The last type is Hybrid-approaches that combine with filter and wrapper method to gain one model.

In recent times, metaheuristic algorithms have been effectively employed in conjunction with various feature selection methods and these have demonstrated successful solutions for various optimization problems, outperforming exact methods^16^. Metaheuristic algorithms can be categorized into two methods: neighborhood-based and population-based^17^. The population-based method explores global optimal features by simultaneously considering multiple points. Population-based algorithms such as differential evolution (DE)^18^, ant colony optimization (ACO)^19^, particle swarm optimization (PSO)^20^, and genetic algorithms (GA)^21^ fall into this category. The neighborhood-based search algorithm focuses on exploring local optimal features by examining a single point at a time. Simulated annealing (SA)^18^ and Tabu search (TS)^22^ are examples of neighborhood-based algorithms.

We proposed a novel model called Voting-Based Enhanced Binary Ebola Optimization Search Algorithm (VBEOSA). It improved the Binary Ebola Optimization Search Algorithm (BEOSA) by combining six classification algorithms using voting model based on lung cancer gene expression dataset. In the context of gene expression data, voting in the BEOSA algorithm provides an additional benefit. Gene expression data analysis often involves high-dimensional datasets with complex relationships. By incorporating multiple classification models and utilizing voting, the BEOSA algorithm can effectively capture the intricate patterns and variability present in gene expression data. The combination of diverse models helps to uncover different aspects of gene expression profiles and improves the interpretation and understanding of gene behavior. This enables more accurate identification of relevant genes and enhances the potential for discovering meaningful biological insights. Therefore, voting in the BEOSA algorithm not only brings diversity, improves accuracy, and increases robustness but also enables better analysis and interpretation of gene expression data.

The paper makes two significant contributions to the field. Firstly, it introduces a novel approach called VBEOSA (Voting Binary Ebola Optimization Search Algorithm), which combines the BEOSA algorithm with a voting model. This integration enhances the feature selection and classification process by leveraging the collective decision-making capabilities of multiple classification models. By applying VBEOSA to gene expression data, which is known for its complexity and high dimensionality, the analysis and interpretation of gene expression profiles are improved, leading to the identification of relevant genes, and providing insights into biological processes. The aim is to achieve an optimal subset of features that maximizes classification models performance while minimizing the number of selected features. This innovative approach improves gene expression analysis and classification accuracy through the integration of the BEOSA algorithm and the voting model. Secondly, the study contributes to the field by leveraging RNASeq gene expression data to identify the differentially expressed genes (DEGs) and discover new biomarkers or hub genes.

The DEGs are further used and analyzed to construct a protein–protein interaction (PPI) network using the STRING database and Cytoscape software. The PPI network captures both direct and indirect interactions. In direct interactions proteins are closely bound together for specific functions. Indirect interactions are known as functional associations. Computational methods and knowledge transfer between organisms are utilized to predict these interactions, incorporating information from primary databases. Additionally, the study conducts Kyoto Encyclopedia of Genes and Genomes (KEGG) pathway and gene ontology (GO) analyses using the Enricher web tool to extract meaningful insights from the DEGs. In addition, we used the weighted gene co-expression network analysis (WGCNA) to identify gene modules that showed strong correlations with clinical characteristics. Additionally, we identified key genes within these selected modules based on their highest connectivity within the respective module. By integrating these approaches, the study aims to identify potential biomarkers and gain a deeper understanding of the biological processes associated with the analyzed gene expression data.

## Related works

The literature contains a wide range of feature selection approaches that make use of metaheuristic optimization methods specifically for gene expression data. Pirgazi et al.^23^ proposed an efficient hybrid filter-wrapper metaheuristic-based gene selection method for high-dimensional datasets. They used different datasets including arcene, colon, prostate1, lung, dfiuse large b-cell lymphoma dataset (DLBCL), Dorothea, Central Nervous System dataset (CNS), prostate, breast, and leukemia. The method combined the strengths of filter and wrapper approaches to select informative genes and improve classification accuracy. It utilized metaheuristic algorithms to search for optimal subsets of genes. The hybrid approach enhanced the efficiency and effectiveness of gene selection, making it suitable for high-dimensional datasets. The experimental results of the proposed algorithm demonstrated its superior accuracy, surpassing similar methods with an average of 93.34%.

A novel approach for attribute selection in lung cancer microarray gene expression data analysis was introduced by Arunkumar and Ramakrishnan^24^. The method employed a customized similarity measure based on fuzzy rough set theory to assess attribute relevance and redundancy. By incorporating information gain and dependency degree, the approach effectively identified the most informative and non-redundant attributes for accurate lung cancer classification. The experimental evaluations using the random forest classifier on gene expression datasets for leukemia, lung, and ovarian cancer yielded accuracies of 86.11%, 81.94%, and 92.89% respectively. A hybrid machine learning framework which combined a nature-inspired cuckoo search (CS) algorithm with genetic algorithm (GA) and artificial bee colony (ABC) was developed by Rabia Musheer Aziz^25^. The framework utilized independent component analysis (ICA) in the preprocessing stage to extract important genes from the dataset. The proposed gene selection algorithms, along with leave-one-out cross-validation (LOOCV) and Naive Bayes (NB) classifier, aimed to identify a small set of informative genes for optimal classification accuracy. The framework's performance was assessed on six benchmark gene expression datasets. Experimental results demonstrated that the ICA and CS-based hybrid algorithm with NB classifier outperformed previously published feature selection methods for the NB classifier.

Oyelade et al.^26^ proposed a novel hybrid binary optimization approach for effective feature selection in high-dimensional datasets. Their approach included a subpopulation selective mechanism that dynamically assigned individuals to a 2-level optimization process. The level-1 method involved mutating population items and then reassigning them to a level-2 optimizer. The selective mechanism determined the subpopulation assigned to the level-2 optimizer based on the exploration and exploitation phase of the level-1 optimizer. Nested transfer (NT) functions were designed and their influence on the level-1 optimizer was investigated. The binary Ebola optimization search algorithm (BEOSA) was used for the level-1 mutation, while the firefly (FFA) and simulated annealing (SA) algorithms were investigated for the level-2 optimizer. The resulting hybrid methods were named HBEOSA-FFA and HBEOSA-SA. Their corresponding variants HBEOSA-SA-NT and HBEOSA-FFA-NT were examined without applying NT. Experimental tests were conducted on high-dimensional datasets to address the challenge of feature selection. The results demonstrated classification accuracies of 0.995 for HBEOSA-FFA on large-scale datasets, 0.967 for HBEOSA-FFA-NT on medium-scale datasets, and 0.953 for HBEOSA-FFA on small-scale datasets.

Akinola et al.^27^ introduced a novel feature selection model called binary Ebola optimization search algorithm (BEOSA). Their proposed model incorporated V-shape and S-shape transfer functions to guide the mutation process in the exploitation and exploration phases. A representation of the binary search space and the mapping from continuous to discrete space were illustrated. The fitness and cost functions used in the algorithm were mathematically formulated. The performance of this method was evaluated on 22 benchmark datasets. The results indicated that the SVM and KNN algorithms performed effectively in conjunction with BEOSA and BIEOSA. The SVM achieved a classification accuracy of 0.845, while the KNN achieved a higher accuracy of 0.935. Bai et al.^28^ introduced a novel approach which combined multi-objective optimization for feature selection and classifier design (JMO-FSCD). Their proposed method incorporated a neural network as the classifier and employed a non-iterative algorithm for training, ensuring efficient performance and rapid learning. To optimize both feature selection and classifier simultaneously, a new coding scheme was devised. To validate the effectiveness of the proposed approach, they compared its performance with six state-of-the-art FS algorithms. Experimental results on thirty-five benchmark datasets demonstrated the superior performance of JMO-FSCD. Yongbin et al.^12^ presented a hybrid feature selection method called HFSIA to address the challenge of feature reduction in high-dimensional data. The proposed model effectively combined the filter method with a metaheuristic-based search strategy. To enhance the search performance of the algorithm they incorporated a Cauchy mutation operator and a lethal mutation mechanism with adaptive adjustment factors. The performance of HFSIA was evaluated through experimental comparisons on 22 high-dimensional benchmark datasets, where it was compared against 23 state-of-the-art feature selection methods. The results indicated that HFSIA achieved a computational cost that was comparable to 5 classical feature selection methods.

Almugren^29^ presented a survey that conducted a thorough examination of hybrid feature selection algorithms used in the analysis of microarray gene expression data for cancer classification. The main objective was to integrate diverse feature selection techniques to identify relevant genes that significantly contribute to accurate cancer classification. Different hybrid models, including combinations of filters, wrappers and embedded methods, were discussed and compared in terms of their limitations, advantages, and performance characteristics. More so, the survey offers a comprehensive overview of hybrid feature selection methods in the analysis of microarray gene expression data for cancer classification. Alhenawi et al.^30^ presented a systematic review centered on the utilization of feature selection models in the analysis of gene expression microarray data for cancer classification. The primary objective was to conduct a thorough and comprehensive analysis of the various feature selection techniques employed in this context. It explored and compared different approaches, including, wrapper, filter and embedded methods. Furthermore, it indicated that the research direction of presented hybrid feature selection algorithms had the highest percentage of 34.9%, suggesting it as the most compelling area of study. Other research directions had lower percentages ranging from 13.6% to 3%. This information serves as a valuable guide for researchers in selecting the most competitive research direction. It considered six key perspectives: methods employed, classifiers used, datasets utilized, range of dataset dimensions, performance metrics evaluated, and the results achieved. A comprehensive overview of hybrid feature selection techniques for analyzing gene expression microarray data in breast cancer was proposed by Mohd et al.^31^. Their work focused more on combining metaheuristic algorithms with feature selection methods to identify the most informative and relevant genes for breast cancer classification. Various metaheuristic approaches such as genetic algorithms, particle swarm optimization, ant colony optimization, and simulated annealing were discussed, highlighting their advantages, limitations, and applications in breast cancer research. Overall, this review provided researchers with valuable insights into the current state-of-the-art hybrid feature selection approaches for breast cancer gene expression microarray data, enabling a comprehensive understanding of the field's advancements.

Elbashir et al.^32^ developed a novel computational technique to identify informative genes for early cancer diagnosis. Through the application of three methods (maximal clique centrality (MCC), maximum neighborhood component (MCN) and node degree), eight common hub genes were identified: ASPM, CDK1, KIF11, TOP2A, AURKB, CCNB2, CENPE, and CCNA2. Enrichment analysis revealed their involvement in various pathways, including focal adhesion, ECM-receptor interaction, melanoma and prostate cancer pathways. Kaplan–Meier survival analysis demonstrated the potential of these hub genes as prognostic and diagnostic biomarkers for breast cancer. Dhirachaikulpanich et al.^33^ utilized microarray and RNASeq data integration to identify age-related macular degeneration (AMD) associated pathways and differentially expressed genes. Their findings revealed two novel pathways: the neuroactive-ligand receptor interaction pathway, and the extracellular matrix (ECM) receptor interaction pathway, which exhibited high enrichment in DEGs related to AMD. Additionally, a protein–protein interaction network analysis identified HDAC1 and CDK1 as central hub genes, involved in regulating cell proliferation and differentiation processes. Hozhabri et al.^34^ conducted an integration analysis of four microarray gene expression datasets related to colorectal cancer (CRC) obtained from the GEO database. They performed differential expression analysis, as well as enrichment analyses for Gene Ontology terms and Kyoto Encyclopedia of Genes and Genomes (KEGG) pathways. The results revealed that the regulation of cell proliferation, bicarbonate transport, Wnt and IL-17 signaling pathways, and nitrogen metabolism were among the most significantly associated pathways with the identified differentially expressed genes.

Luo et al.^35^ employed a comprehensive approach that involved the identification of overlapping genes between DEGs and WGCNA, leading to subsequent GO and KEGG analyses. This method allowed them to identify hub genes, which were then subjected to survival analysis. Interestingly, among the ten hub genes, only SPP1 demonstrated a significant impact on lung cancer survival. The authors further delved into the analysis of SPP1, predicting associated miRNAs and lncRNAs, which were subsequently utilized for a rigorous survival analysis. In a related study, Niemira et al.^36^ leveraged WGCNA to explore molecular networks associated with a range of clinical traits, including tumor size, SUVmax, BMI, smoking status, recurrence-free survival, and disease-free survival. Their findings highlighted the significance of a more profound investigation into the identified genes and pathways, particularly those linked to the tumor microenvironment and mechanisms related to immune evasion in adenocarcinoma (ADC) and squamous cell carcinoma (SCC). Furthermore, they constructed a protein–protein interaction network of the DEGs using Cytoscape software, leading to the identification of key hub genes, such as MYC, CXCL1, CD44, MMP1, and CXCL12. Nisar et al.^37^ conducted an integration analysis of microarray and RNASeq gene expression data in the context of pancreatic cancer (PaCa) to unearth differentially expressed genes. Their approach included a protein–protein interaction (PPI) network analysis and pathway investigations. Their results shed light on the significance of hub genes, including ITGA1, ITGA2, ITGB1, ITGB3, MET, LAMB1, VEGFA, PTK2, and TGFb1 in PaCa. Moreover, their analysis revealed two critical pathways, namely the ECM-receptor interaction and focal adhesion pathways, which play crucial roles in the development and progression of PaCa. For a concise overview of related work in the literature, please refer to Table 1, which summarizes these significant findings.

**Table 1:** Comparative summary of related existing studies.

Authors	Year	Method	Results	Limitation
Pirgazi et al.^23^	2019	hybrid filter-wrapper metaheuristic	Accuracy of 93.34%	The study used small samples
Arunkumar and Ramakrishnan^24^	2018	customized similarity measure based on fuzzy rough set theory	Accuracy of 81.94%	The study did not use a combination machine learning classifier and metaheuristic-based hyperparameter optimizers
Rabia Musheer Aziz^25^	2022	CS, GA, and ABC	Accuracy of 99.21%	They didn’t use different classifiers
Akinola et al.^27^	2022	BEOSA	Accuracy of 0.935	They didn’t use voting to combine the performance of all different classifiers
Bai et al.^28^	2023	JMO-FSCD	Accuracy of 96.78%	They didn’t use different classifiers, and they used small samples
Dhirachaikulpanich et al.^33^	2020	AMD	Two key hub genes were identified	The study did not use a combination machine learning classifier and metaheuristic-based hyperparameter optimizers
Luo et al.^35^	2021	WGCNA, GO, and KEGG	SPP1 was correlated with lung cancer	The study did not use a combination machine learning classifier and metaheuristic-based hyperparameter optimizers
Niemira, et al.^36^	2019	WGCNA	top hub genes in modules associated	The study used a relatively small number of samples

## Material and methods

### Dataset and pre-processing

**Figure 1:**
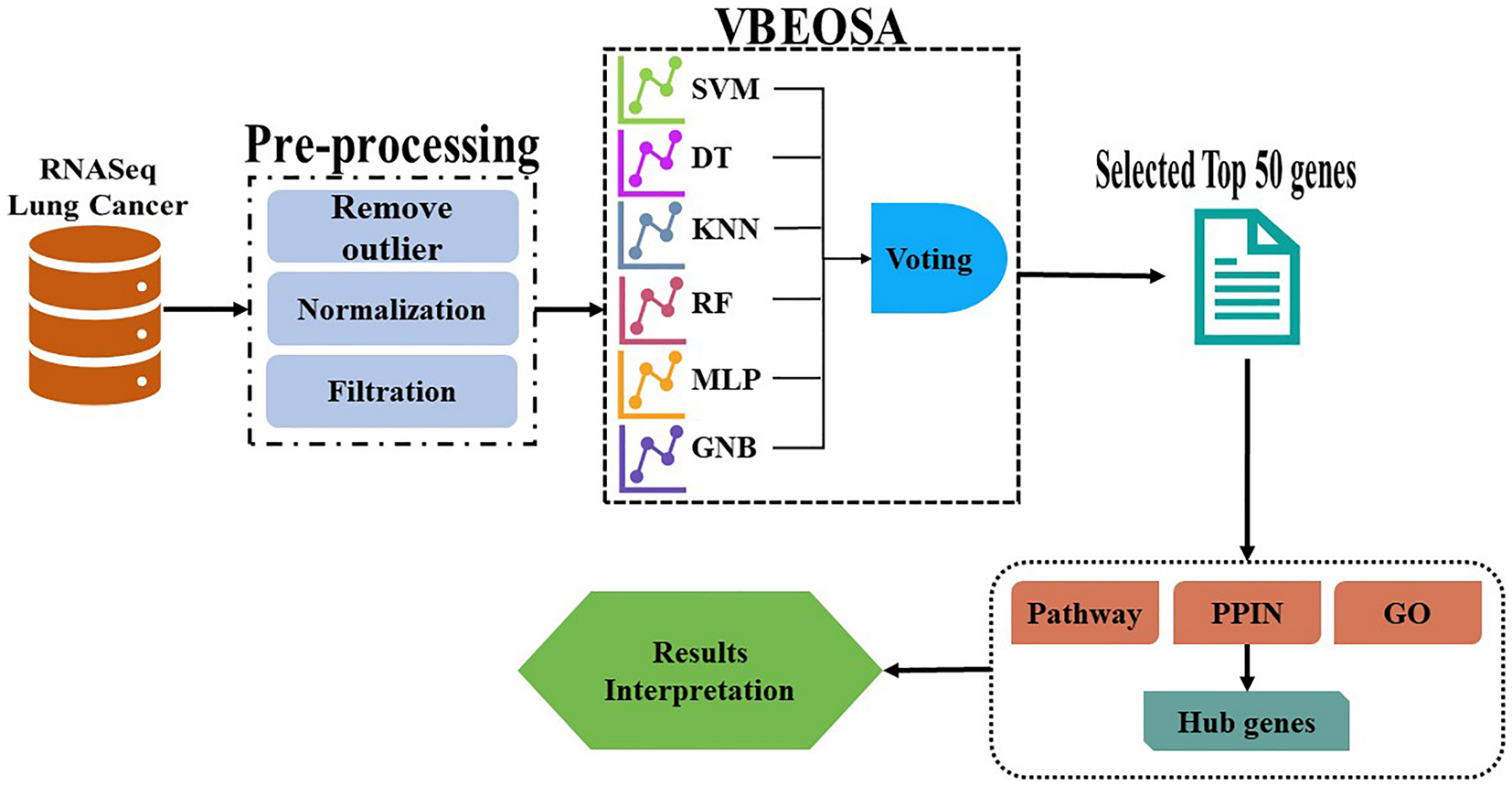
The proposed methodology.

We utilized the R software to analyze the lung cancer gene expression data obtained from the Cancer Genome Atlas (TCGA) repository (https://portal.gdc.cancer.gov/) The GDCquery function, available in the TCGAbiolinks library, was employed to query the data^38–41^. The lung cancer dataset consisted of 1208 clinical samples and 14,895 genes or features. Among these, there were 113 paracancerous normal tissues and 1095 tumor samples. Due to the presence of noise and numerous other features, various pre-processing steps namely, outlier removal, normalization, and filtration were implemented to obtain clean data that specifically contributed to lung cancer detection. To identify outlier samples, we calculated the array-array intensity correlation (AAIC), which measures the Spearman correlation between samples^39^. Using a cutoff value of 0.6, samples exceeding this threshold were considered outliers and removed from the analysis. Normalization was applied to the gene expression data to ensure the accuracy of expression levels and eliminate biases in the analysis. The TCGAanalyze-Normalization function from the TCGAbiolinks library was employed for this purpose. Subsequently, filtration was performed by selecting genes with mean expression values above a cutoff value of 0.25, resulting in a reduction in the number of genes^39^. As a result of these pre-processing steps, the dataset consisted of 1208 clinical samples and 14,895 genes. Figure 1 shows the proposed methodology.

### VBEOSA model

Following the completion of the pre-processing step, the pre-processed dataset was utilized as input for the VBEOSA model to identify the top 50 genes that exhibited high significance. A recent metaheuristic algorithm BEOSA^27^ is derived from the EOSA metaheuristics. EOSA itself is inspired by the infection mechanism of the Ebola virus and utilizes a binary optimization approach^42^. BEOSA aims to address feature selection and classification problems and it involves two main stages: initialization and optimization. In the initialization stage, an initial population of candidate solutions was generated. Then, in the optimization stage, the algorithm iteratively updates the population by performing selection, mutation, and crossover operations. Fitness evaluation was performed using a classification algorithm, including support vector machines (SVM), decision tree (DT), k-nearest neighbors (KNN), random forest (RF), multi-layer perceptron (MLP), and Gaussian Naïve Bayes (GNB). The VBEOSA (Voting Binary Ebola Optimization Search Algorithm) model is an enhanced version of the BEOSA algorithm that incorporates a voting mechanism to improve feature selection and classification performance. It leverages the collective decision-making capabilities of multiple classification models. The VBEOSA model follows a similar iterative process as the BEOSA algorithm, but with the addition of a voting step. Initially, a population of binary strings is initialized, representing potential subsets of features. The fitness of each binary string is evaluated by applying multiple classification models to the corresponding feature subset. This evaluation is based on performance metrics.

Ensemble-based classifiers are meta-classifiers that combine multiple machine learning classifiers for classification tasks. They utilize either hard voting, which involves selecting the majority prediction from the individual classifiers, or soft voting, which averages the class probabilities predicted by each classifier. Hard voting relies on the majority vote to make the final prediction^43,44^. Soft voting was employed to combine the classification models in this study. Soft voting involves averaging the class probabilities predicted by each classification model to make the final prediction. By considering the aggregated probabilities, the soft voting approach leverages the strengths and expertise of each individual model, leading to improved classification accuracy and more robust predictions. The voting mechanism is then applied to determine the overall fitness of each binary string. This can involve a majority voting scheme, where each classification model's prediction contributes to the final decision, or weighted voting that assigns more weight to certain models based on their performance. The binary strings with higher fitness, determined by the voting mechanism, are selected for the next generation. Genetic operators like crossover and mutation are applied to the selected binary strings to generate offspring, promoting diversity and exploration. The newly generated offspring replace some of the original binary strings in the population. This process continues for a specified number of iterations or until a termination criterion is met.

The VBEOSA model combines the predictions from multiple classification models using the voting mechanism, which allows for collective decision-making. By integrating the voting mechanism into the BEOSA algorithm, the VBEOSA model enhances feature selection accuracy and robustness. It improves the identification of relevant features by considering the consensus among multiple classification models. The selected features can be used for subsequent analysis or classification tasks. In summary, the VBEOSA model provides an innovative approach to feature selection by leveraging the power of multiple classification models through a voting mechanism. It enhances the analysis and interpretation of gene expression data by selecting informative features and improving classification accuracy. The algorithm listing 1 represents the pseudocode for VBEOSA algorithmic design steps.

The algorithm was presented through the flowchart outlined in this subsection. First, we introduce the algorithmic formalization, as shown in Algorithm listing 1. The algorithm requires input values for $epo$ (the maximum number of iterations), $popsiz$(population size), $serate$ (short-distance rate), and $lerate$ (long-displacement rate). In return, it provides the global best solution, the cost values for each iteration, and the count of features obtained during the optimization process. The algorithm achieves binarization of the solution space and calculates fitness values for each solution in Lines 4–5. Subsequently, it then computes the current global best solution and the displacement positions for all individuals in the susceptible compartment as shown in Lines 6–7. The iteration for the optimization process is outlined in Lines 8–38, contingent upon two conditions: first, that the maximum number of iterations has not been reached, and second, that some individuals remain infected. The number of individuals to be quarantined from the infected population is estimated, and a clear demarcation between quarantined and infected individuals is established in Lines 9–10. Iteration of the infected individuals is defined in Line 11, and the number of newly infected cases in the susceptible group is depicted in Line 12. In Lines 13–28, we iterate through the newly infected cases and generate the discriminant value in Line 14. If the condition in Line 15 is met, it implies that the method will explore a local space; otherwise, it will explore a global space. In both cases of exploitation and exploration, we compute the expected number of infections. In Lines 17–21, we used either the $S1()$ or $S2()$ function based on the value of $d$. Furthermore, depending on the condition in Line 18, the feature position in that individual is mutated to either 1 or 0. A similar procedure is repeated for the exploration phase, utilizing either the $T1$ or $T2$ function depending on the value of $d$. Finally, the compartments are updated, and the global best solution is determined before proceeding to the next iteration.

**Algorithm 1:**
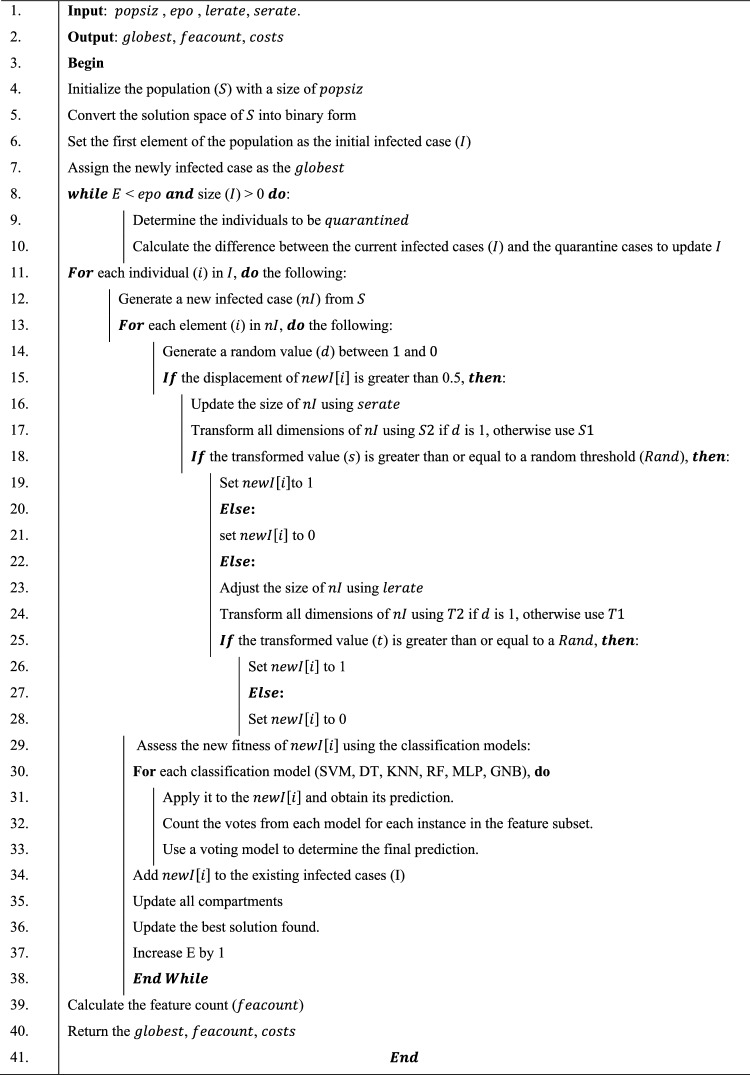
Pseudocode VBEOSA algorithm.

### Mathematical model

To facilitate the discussion of the proposed VBEOSA method, we provide a summary of the mathematical models used in the techniques. The population initialization of EOSA is represented by Eq. (1), as follow:

1 $$indi = L +rand*\left( U- L\right)$$

In the context of the optimization problem, rand represents a randomly generated real number, while L and U denote the lower and upper bounds, respectively. Equation (2) describes the mutation process of infected individuals in the continuous space, where $\Delta$ represents the change factor of an individual and $gbest$ represents the $global$ best solution.

2 $${ind i}^{new} = \Delta *{e }^{rand}\mathit{cos}\left(2\pi rand\right)*(indi- gbest)$$

References^45,46^ provide detailed calculations for the allocation of individuals to compartments Q (Quarantine), S (Susceptible)), R (Recovered), V (Vaccinated), I (Infected), H (Hospitalized), and D (Dead).

BEOSA introduced four transformation functions to locate infected individuals in the discrete space. These functions are categorized as S-functions and V-functions, with two functions belonging to each category. Equations (3) and (4) define the $S1$ and $S2$ functions, which are part of the S-transform function family. On the other hand, Eqs. (5) and (6) represent the $V1$ and $V2$ functions, which are part of the V-function family.

3 $$S1=\frac{1}{1+{e}^{(\frac{-x}{2})}}$$

4 $$S2=1-\frac{1}{1+{e}^{x}}$$

5 $$V1=\left|\frac{x}{\sqrt{2+{x}^{2}}}\right|$$

6 $$V2=\left|tanx\right|$$

The purpose of applying these transform functions is to facilitate the conversion of feature positions within an individual to either 0 or 1. These functions also enhance the likelihood of altering the original composition of the individual, making it a potential solution for feature selection problems. This concept is demonstrated through Eqs. (4) and (6). The initial segment of the equations determines whether the $S1$ or $S2$ function is used for the S-function, or whether the $T1$ or $T2$ function is used for the V-function. A determinant factor guides this decision, where a random number, rand ($0|1$), is compared to a threshold. If rand ($0|1$) is greater than the threshold, the $S2$ or $T2$ function is called; otherwise, the $S1$ or $T1$ function is utilized. In the latter portion of the equations, the value at the kth position in the representation of the individual, indi, is modified to 1 when r is greater than S—indk i, for S-functions, or T—indk i, for T-functions. Conversely, when r is below this threshold, the kth position is assigned a value of 0. Here, k ranges from 0 to D, and r is a randomly generated number between 0 and 1.

The calculation of the fitness and cost functions in this paper depends heavily on the classification accuracy achieved by various classifiers when applied to a selected part of the dataset. Moreover, the main goal of the study is to analyze the effect of different widely used classifiers on solving the feature selection problems such as K-nearest-neighbor (KNN), random forest (RF), multi-layer perceptron (MLP), decision tree (DT), support vector machine (SVM), Gaussian Naive Bayes (GNB), and soft voting. These classifiers were explored to assess their efficacy in addressing the feature selection challenge. Based on this approach, the number of features selected for a random individual, denoted as ${1}^{{IND}_{i}^{k}}$, is calculated using Eq. (7). In this equation, $D$ represents the dimension of the feature size within the dataset, and '1indik' signifies the count of feature positions with a value of 1 in the individual ${1}^{{INK}_{i}^{k}}$. The calculation can be expressed as follows:

7 $$Fci =\left({\sum }_{K=0}^{D}\left({1}^{{IND}_{i}^{k}}\right)\right)\div D$$

This equation essentially sums up the count of feature positions with a value of 1 in ${IND}_{i}$ across all dimensions $D$. It quantifies the number of features that are selected for that individual based on its binary representation. This process illustrates how the study determined the feature selection.

#### Classification metrics

In order to assess the effectiveness of our model, we conducted a comprehensive evaluation using a range of performance metrics, which encompass classification accuracy, precision, recall, F1-score, and the area under the curve (AUC). In Eqs. (8)–(12), the terms False Positives (FP) are instances in which the model incorrectly predicts samples as cancerous when they are actually not, True Positives (TP) represent the count of correctly identified cancerous samples, False Negatives (FN) are cases in which cancerous images are erroneously classified as non-cancerous, True Negatives (TN) indicate the number of non-cancerous samples accurately classified as such. The following Eqs. (8) to (12) outline the definitions of these key metrics.

8 $$Acuraccy =\frac{True\_Positives+True\_Negatives}{True\_Positives+ True\_Negative+ False\_Negatives+ False\_Positives}$$

9 $$Recall=\frac{True\_Positive}{True\_Positives + False\_Negatives}$$

10 $$Precision=\frac{True\_Positives}{\left(True\_Positives + False\_Positives\right)}$$

11 $$F1Score=\frac{2*\left(Recall*Precision\right)}{\left(Recall+Precision\right)}$$

12 $$AUC=\frac{1}{2}\left(\frac{True\_Positives}{True\_Positives + False\_Negatives}+ \frac{True\_Negatives}{True\_Negatives +False\_Positives}\right)$$

### Gene ontology (GO) and pathway enrichment analysis

Biological functions common to cells or organisms with a well-defined nucleus are determined by a considerable portion of genes. If the biological function of a shared protein is known, it can be transferred from one organism to another. The Gene Ontology resource (GO) (http://geneontology.org) offers structured and computable information about gene and gene product functions. Established in 1998, GO has gained significant recognition in the life sciences field and is constantly evolving in terms of its content, encompassing both increased quantity and improved quality^47^. The GO is responsible for identifying the biological process, molecular function, and cellular location associated with an organism's genes. It comprises two main components: the GO annotation and the ontology. The ontology is structured as a hierarchical tree of concepts called GO terms. The GO annotation refers to the list of all annotated genes that are associated with ontology terms, providing descriptions for these genes^48^.

To identify the gene functionality associated with various pathways and transcription factors regulating the expression of other genes, genes enrichment analysis is performed. The analysis utilizes a list of common genes as input and compares it with pre-existing gene-set libraries containing prior knowledge. The enrichR web server, developed by the Ma'ayan lab, is employed for this analysis. This open-source web-based gene enrichment analysis tool integrates results from multiple gene-set libraries. The KEGG pathway database is used to identify pathways related to the common DEGs list. Significance of pathways is determined using the Fisher's exact test p-value, with a threshold of < 0.05, and a high combined score^49^.

### Analysis of protein–protein interactions (PPI) network

Protein–Protein Interaction (PPI) Network Analysis is performed using the STRING biological database, which integrates information from diverse sources to predict functional interactions among proteins^50,51^. Known and predicted PPI data from STRING is utilized to identify potential interactions among the DEGs. The resulting PPI network is then analyzed and visualized using Cytoscape software. To enhance network visualization and mitigate the complexity known as the "hairball effect"; a simplified zero-order interaction network is constructed. NetworkAnalyzer software is employed to calculate important network properties such as degree distribution, clustering coefficients, and centrality measures^52^. The degree of a node represents the number of connections it has with other nodes, while betweenness centrality indicates the number of shortest paths between a node and other highly connected nodes.

### Weighted gene co-expression network analysis

We utilized the WGCNA package in R to establish co-expressed gene modules and, notably, identified a significant gene module with the most robust correlation to lung cancer, as documented in Refs.^35,53,54^. Furthermore, we applied WGCNA to the selected genes to explore the intricate expression patterns that existed among them. Genes displaying strong interrelationships within the network were thoughtfully grouped into distinct clusters, effectively giving rise to specific modules. These modules represent assemblies of genes exhibiting highly correlated expression patterns within an unsigned co-expression network. To facilitate their differentiation, genes with similar expression patterns were visually distinguished by the assignment of unique colors, as elaborated in references^55,56^.

## Results and discussion

### VBEOSA model results

**Figure 2:**
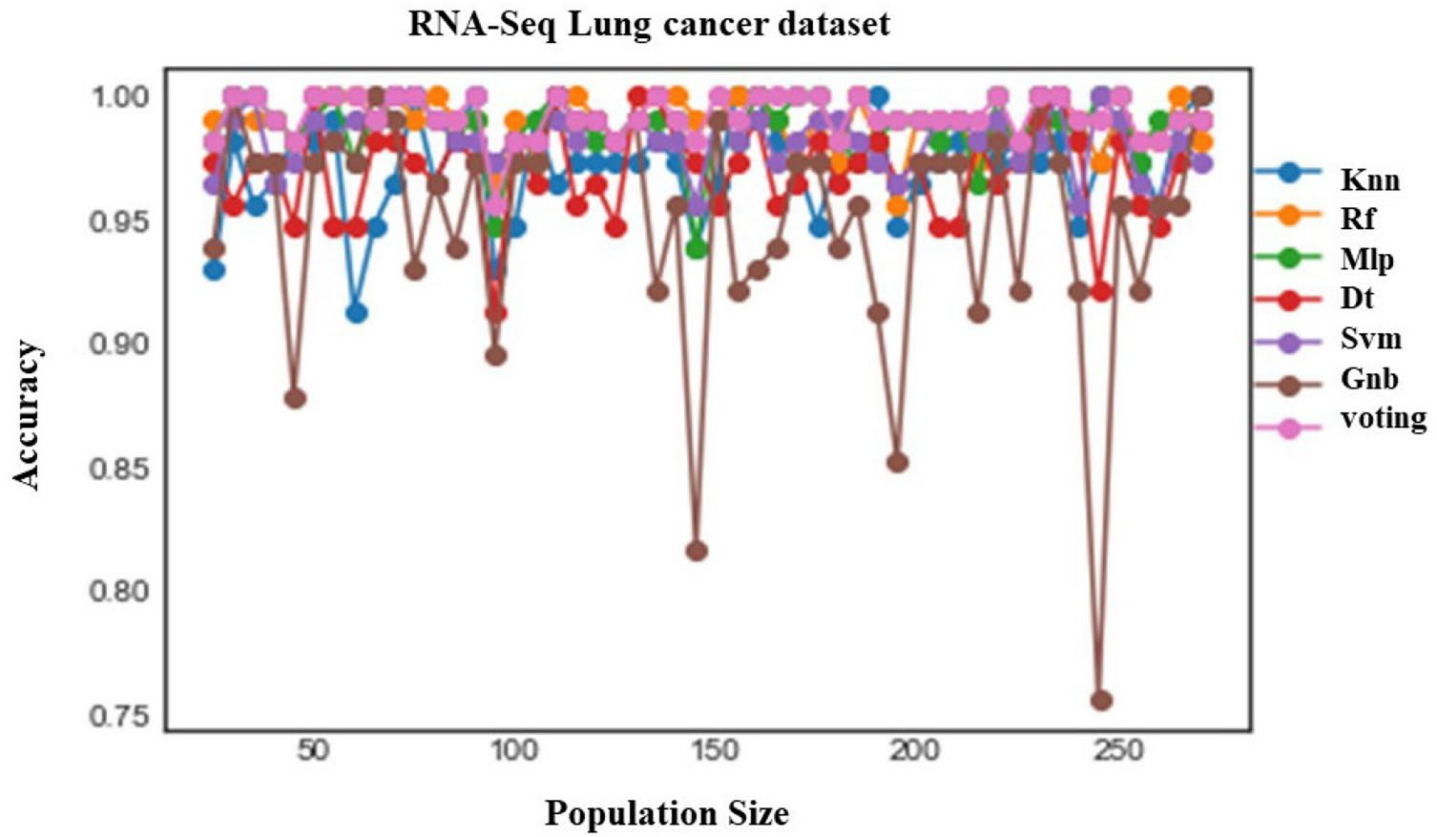
The accuracy of models based on 50 population size.

In our experiments we utilized VBEOSA with specific parameter settings. The values assigned to the parameters ${\varvec{\pi}}$, ${\varvec{\beta}}1$, ${\varvec{\beta}}2$, ${\varvec{\beta}}3$, and ${\varvec{\beta}}4$ were uniformly set to 0.1. Additionally, we employed a population size of 50, ranging from 25 to 270 individuals with an increment of 5. In our quest to identify the top 50 genes, we conducted a comprehensive evaluation of various classification models, including KNN, RF, MLP, DT, SVM, Naïve Bayes, and voting. To facilitate this analysis, we utilized VBEOSA with population sizes ranging from 25 to 270. Our findings revealed that the voting model consistently exhibited the highest accuracy when compared to the other models under consideration (see Fig. 2). Table 2 displays the initial results of classification metrics for the Lung cancer dataset using DT, GNB, KNN, MLP, RF, SVM, and voting model. On the other hand, Table 3 provides a comparison between our proposed VBEOSA and other implemented binary algorithms, namely, BWOA, BDMO, and BSNDO, with the voting mechanism applied to each respective algorithm. Our proposed model demonstrated superior performance compared to other methods, achieving a precision of 0.99195, recall of 0.99106, F-measure of 0.98881, and an AUC of 0.98000. However, it is noteworthy that the accuracy of BWOA (0.98200) and BSNDO (0.98526) surpasses the accuracy of our proposed model (0.97985). It is crucial to acknowledge that accuracy may not always provide a comprehensive depiction of a model's overall performance.

**Table 2:** Classification metrics results for the Lung cancer dataset on six meta-classifiers namely, DT, GNB, KNN, MLP, RF, and SVM without voting mechanism.

Model	Voting	DT	SVM	GNB	KNN	MLP	RF
Accuracy	**0.98200**	0.98196	0.98198	0.98199	0.98193	0.98196	0.98195
Precision	**0.98966**	0.98948	0.98948	0.98948	0.98948	0.98951	0.98951
Recall	**0.98890**	0.98593	0.98596	0.98596	0.98584	0.98593	0.98593
F1-Score	**0.98872**	0.98380	0.98385	0.98383	0.98375	0.98380	0.98380
AUC	**0.97924**	0.96805	0.96807	0.96806	0.96805	0.96818	0.96820

Significant values are in bold.

**Table 3:** Classification metrics results for the Lung cancer dataset on the VBEOSA, BWOA, BDMO, and BSNDO algorithms with voting mechanism.

Model	VBEOSA	BWOA	BDMO	BSNDO
Accuracy	0.97985	0.98200	0.97528	0.98526
Precision	**0.99195**	0.98966	0.98584	0.99133
Recall	**0.99106**	0.98387	0.98038	0.98890
F1-Score	**0.98881**	0.98374	0.97959	0.98631
AUC	**0.98000**	0.96873	0.96873	0.97125

Significant values are in bold.

In our particular scenario, the gene expression data related to lung cancer exhibits notable noise and a significant imbalance in the distribution of tumors to the paracancerous normal tissues, resulting in one class having considerably more instances than the other. Despite these inherent challenges, our proposed model demonstrated robust overall performance when compared to other algorithms. To evaluate the model's effectiveness in handling gene expression data with common imbalance issues, we employed various classification metrics, including precision, recall, F1-score, and AUC. Furthermore, we implemented a voting approach as a meta-classifier, utilizing DT, GNB, KNN, MLP, RF, and SVM as our selected base learners. The outputs generated by these base learners were aggregated and passed to the meta-classifier, enhancing the model's ability to make final predictions and, in turn, improving its capacity to generalize effectively.

### The analysis of Gene Ontology (GO) on the common differentially expressed genes (DEGs)

The GO term analysis was conducted to investigate the functional characteristics and biological processes associated with the common differentially expressed genes (DEGs). By performing this analysis, we aimed to gain insights into the functional roles and molecular functions of the DEGs in the studied phenomenon. The analysis helps to identify enriched Gene Ontology (GO) terms that provide information about the biological processes, molecular functions, and cellular components related to the DEGs. This analysis enhances our understanding of the underlying biological mechanisms and pathways involved in the observed gene expression changes. We used Fisher's exact test to rank the genes based on their p-values, indicating the probability of each gene belonging to one of the GO term categories.

The analysis of enriched GO terms in the biological process category reveals that our DEGs are significantly enriched and related to Vasodilation (GO:0,042,311, P-value = 0.000006159), Adenylate Cyclase Activating Adrenergic Receptor Signaling Pathway (GO:0,071,880, P-value = 0.000006159), Positive Regulation Of Adenylate Cyclase Activity (GO:0,045,762, P-value = 4.568e − 7), Positive Regulation Of Protein Kinase A Signaling (GO:0,010,739, P-value = 0.0002094), Adrenergic Receptor Signaling Pathway (GO:0,071,875, p-value = 0.00001099), Activation Of Adenylate Cyclase Activity (GO:0,007,190, P-value = 0.00001530), Desensitization Of G Protein-Coupled Receptor Signaling Pathway (GO:0,002,029, P-value = 0.0003189), Regulation Of Circadian Sleep/Wake Cycle, Sleep (GO:0,045,187, P-value = 0.01219), Positive Regulation Of Necroptotic Process (GO:0,060,545, P-value = 0.01219), and Positive Regulation Of Programmed Necrotic Cell Death (GO:0,062,100, P-value = 0.01219) (see Table 4).

**Table 4:** The biological process group exhibited significant enrichment of the top 10 Gene GO terms among the DEGs.

GO term	P-value	Odds ratio	Combined score
Vasodilation (GO:0,042,311)	0.000006159	108.36	1300.11
Adenylate cyclase activating adrenergic receptor signaling pathway (GO:0,071,880)	0.000006159	108.36	1300.11
Positive regulation of adenylate cyclase activity (GO:0,045,762)	4.568e-7	80.52	1175.54
Positive regulation of protein kinase A signaling (GO:0,010,739)	0.0002094	121.24	1027.07
Adrenergic receptor signaling pathway (GO:0,071,875)	0.00001099	86.68	989.75
Activation of adenylate cyclase activity (GO:0,007,190)	0.00001530	76.47	847.92
Desensitization of G protein-coupled receptor signaling pathway (GO:0,002,029)	0.0003189	94.29	759.09
Regulation of circadian sleep/wake cycle, sleep (GO:0,045,187)	0.01219	103.89	457.85
Positive regulation of necroptotic process (GO:0,060,545)	0.01219	103.89	457.85
Positive regulation of programmed necrotic cell death (GO:0,062,100)	0.01219	103.89	457.85

The enriched GO term results in the cellular components category were further investigated, and they were found to be highly significant and associated with our DEGs. These cellular components are: Membrane Attack Complex (GO:0,005,579, P-value = 0.01461), Gap Junction (GO:0,005,921, P-value = 0.001199), Pseudopodium (GO:0,031,143, P-value = 0.01944), PRC1 Complex (GO:0,035,102, P-value = 0.03614), Desmosome (GO:0,030,057, P-value = 0.04086), Connexin Complex (GO:0,005,922, P-value = 0.04321), Ficolin-1-Rich Granule (GO:0,101,002, P-value = 0.001063), Endocytic Vesicle Lumen (GO:0,071,682, P-value = 0.05023), Ficolin-1-Rich Granule Lumen (GO:1,904,813, P-value = 0.003403), and Actin Filament (GO:0,005,884, P-value = 0.01312) (see Table 5).

**Table 5:** The cellular component group exhibited significant enrichment of the DEGs in the top 10 enriched GO terms.

GO term	P-value	Odds ratio	Combined score
Membrane attack complex (GO:0,005,579)	0.01461	83.11	351.21
Gap junction (GO:0,005,921)	0.001199	44.64	300.28
Pseudopodium (GO:0,031,143)	0.01944	59.36	233.90
PRC1 complex (GO:0,035,102)	0.03614	29.67	98.51
Desmosome (GO:0,030,057)	0.04086	25.96	83.00
Connexin complex (GO:0,005,922)	0.04321	24.43	76.75
Ficolin-1-rich granule (GO:0,101,002)	0.001063	9.76	66.84
Endocytic vesicle lumen (GO:0,071,682)	0.05023	20.76	62.10
Ficolin-1-rich granule lumen (GO:1,904,813)	0.003403	10.78	61.25
Actin filament (GO:0,005,884)	0.01312	12.26	53.14

By exploring the results of enriched GO terms in the molecular function category, we found significant enrichment of our DEGs, indicating their association with N-acylsphingosine Amidohydrolase Activity (GO:0,017,040, P-value = 1 0.01219), Alcohol Dehydrogenase Activity, Zinc-Dependent (GO:0,004,024, P-value = 2 0.01461), RNA Polymerase III Type 3 Promoter Sequence-Specific DNA Binding (GO:0,001,006, P-value = 0.01461), RNA Polymerase III Cis-Regulatory Region Sequence-Specific DNA Binding (GO:0,000,992, P-value = 0.01703), Alcohol Dehydrogenase (NAD +) Activity (GO:0,004,022, P-value = 0.01944), Acting On Carbon–Nitrogen (But Not Peptide) Bonds, In Linear Amides (GO:0,016,811, P-value = 0.0003496), Water Channel Activity (GO:0,015,250, P-value = 0.03377), Adrenergic Receptor Binding (GO:0,031,690, P-value = 0.03614), CoA Hydrolase Activity (GO:0,016,289, P-value = 0.03614), and Water Transmembrane Transporter Activity (GO:0,005,372, P-value = 0.03850) (see Table 6).

**Table 6:** In the molecular function group, we observed significant enrichment of the DEGs in the top 10 enriched GO terms.

GO term	P-value	Odds ratio	Combined score
N-acylsphingosine amidohydrolase activity (GO:0,017,040)	1 0.01219	103.89	457.85
Alcohol dehydrogenase activity, zinc-dependent (GO:0,004,024)	2 0.01461	83.11	351.21
RNA polymerase III type 3 promoter sequence-specific DNA binding (GO:0,001,006)	0.01461	83.11	351.21
RNA polymerase III Cis-regulatory region sequence-specific DNA binding (GO:0,000,992)	0.01703	69.25	282.07
Alcohol dehydrogenase (NAD +) activity (GO:0,004,022)	0.01944	59.36	233.90
Hydrolase activity, acting on carbon–nitrogen (but not peptide) bonds, in linear amides (GO:0,016,811)	0.0003496	24.48	194.86
Water channel activity (GO:0,015,250)	0.03377	31.95	108.26
Adrenergic receptor binding (GO:0,031,690)	0.03614	29.67	98.51
CoA hydrolase activity (GO:0,016,289)	0.03614	29.67	98.51
Water transmembrane transporter activity (GO:0,005,372)	0.03850	27.69	90.18

### The KEGG pathway enrichment analysis for the DEGs

The Enrichr R package was utilized to identify the top 10 lung cancer pathways associated with the significant DEGs. The pathways identified include Salivary secretion, Dilated cardiomyopathy, Renin secretion, Arrhythmogenic right ventricular cardiomyopathy, Vibrio cholerae infection, cGMP-PKG signaling pathway, Regulation of lipolysis in adipocytes, Vascular smooth muscle contraction, Calcium signaling pathway, and Circadian entrainment. These pathways are considered to be the most significant in relation to lung cancer (see Table 7).

**Table 7:** Top 10 pathways.

GO term	P-value	Odds ratio	Combined score
Salivary secretion	0.000003164	25.65	324.82
Dilated cardiomyopathy	0.000003700	24.80	310.18
Renin secretion	0.00002445	27.19	288.78
Arrhythmogenic right ventricular cardiomyopathy	0.00003772	24.20	246.53
Vibrio cholerae infection	0.0002498	27.62	229.10
cGMP-PKG signaling pathway	0.000003218	17.15	216.91
Regulation of lipolysis in adipocytes	0.0003315	24.96	199.95
Vascular smooth muscle contraction	0.00001818	17.60	192.09
Calcium signaling pathway	0.000001836	14.10	186.29
Circadian entrainment	0.00009318	18.98	176.16

### PPI network and selecting hub genes results

**Figure 3:**
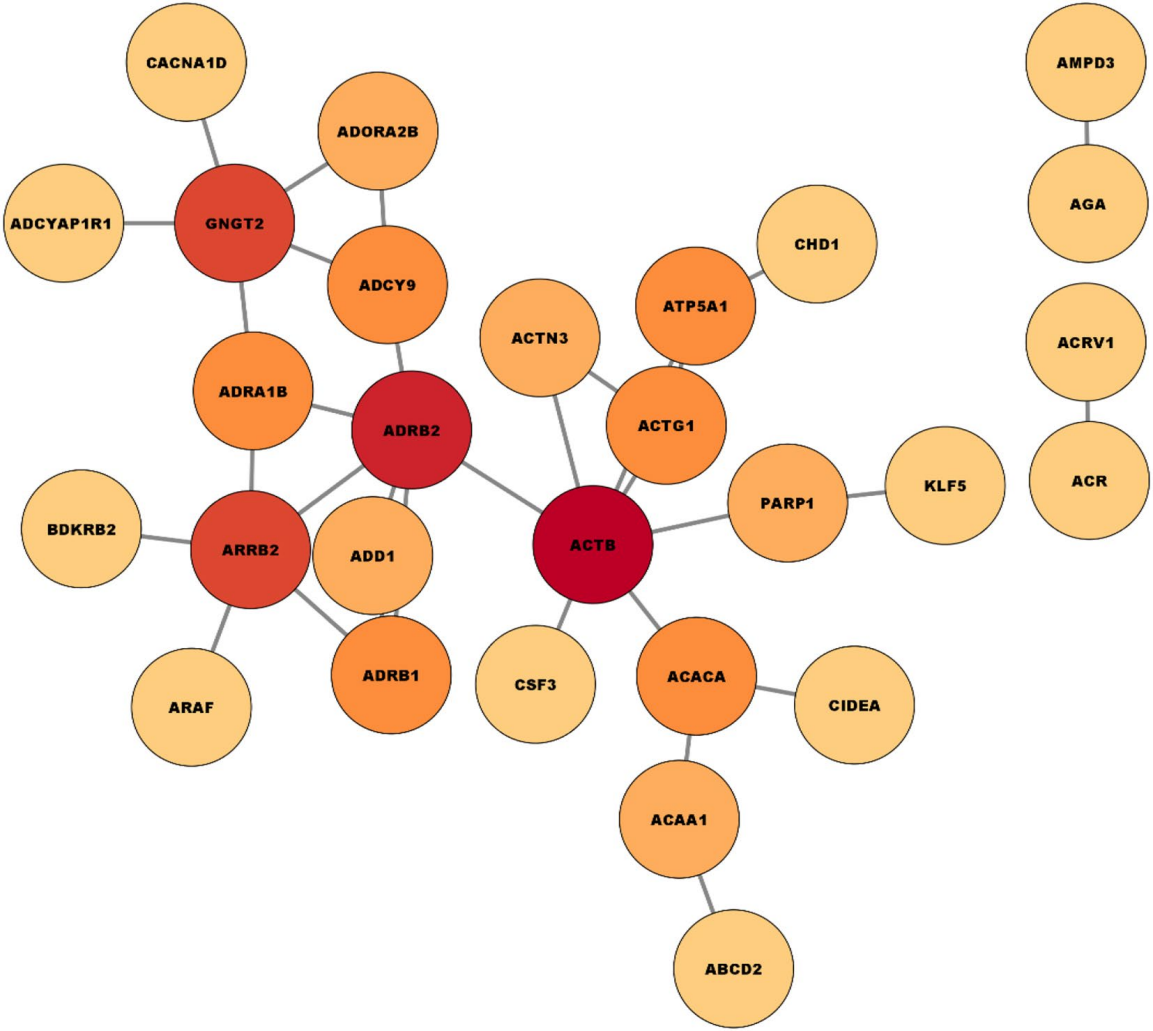
Complete PPIN of differentially expressed genes in lung cancer.

**Figure 4:**
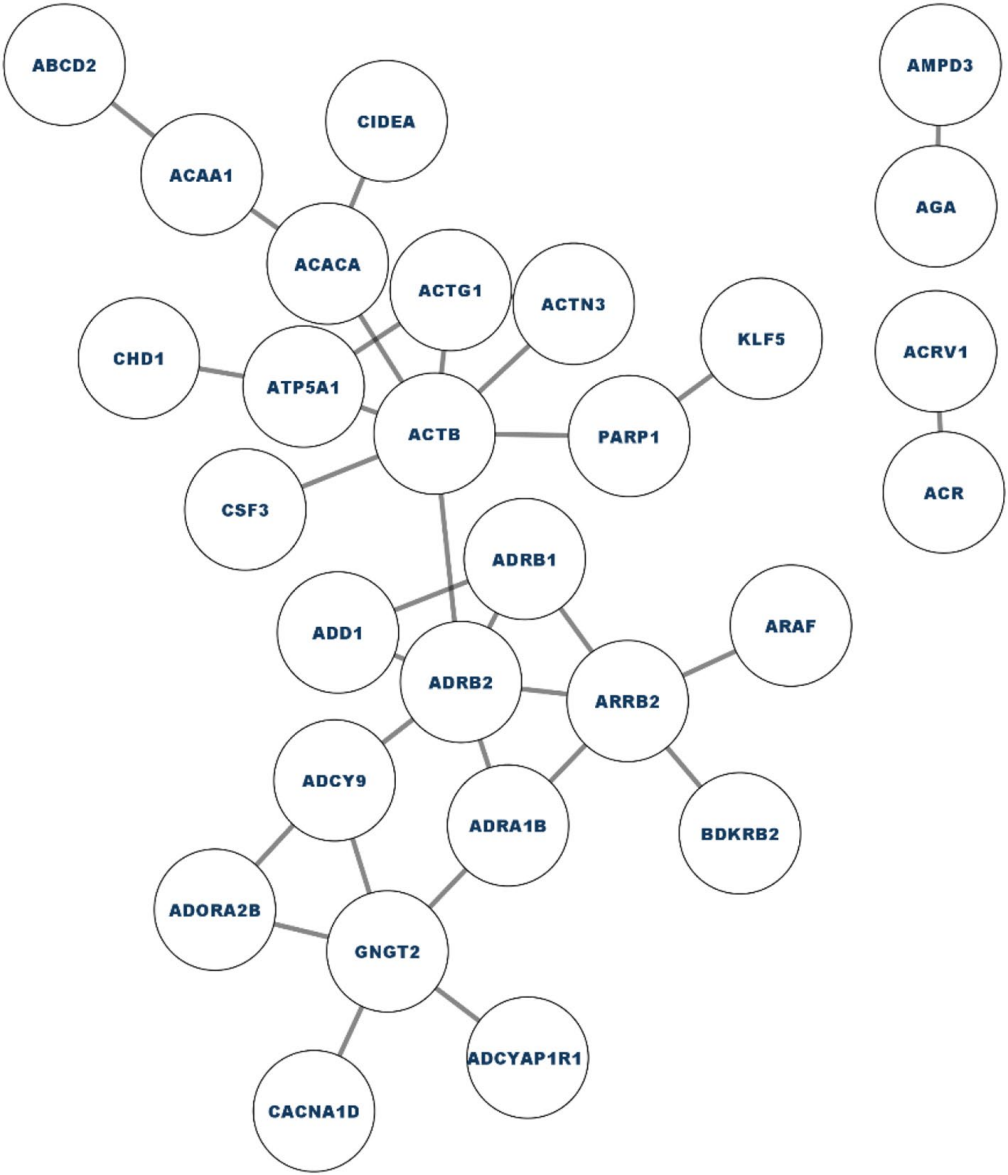
Zero order PPI.

To gain a deeper understanding of our list of differentially expressed genes (DEGs), we conducted further investigations by exploring them in a protein–protein interaction (PPI) network. The PPI network was constructed using the STRING database and Cytoscape application, initially a first-order network was created, which resulted in a large network consisting of 49 nodes and 32 edges (see Fig. 3). Nodes with a dark red color represent a high degree, while nodes with a light red color indicate a low degree. However, due to the size of the network, it was challenging to visualize and focus on the important nodes. To address this, a zero-order PPI network was constructed (see Fig. 4), This led to the formation of a more focused and simplified network, where each node had at least one connection. Notably, ADRB2, ACTB and ARRB2 were among the significant nodes in the network. Additionally, the hub genes were identified using the maximal clique centrality (MCC) method, implemented through the CytoHubba plugin in Cytoscape. The top 10 genes with the highest MCC scores were designated as hub genes. An identified hub gene network was observed in the analysis. This network comprises key genes that exhibit a central role in lung cancer.

**Figure 5:**
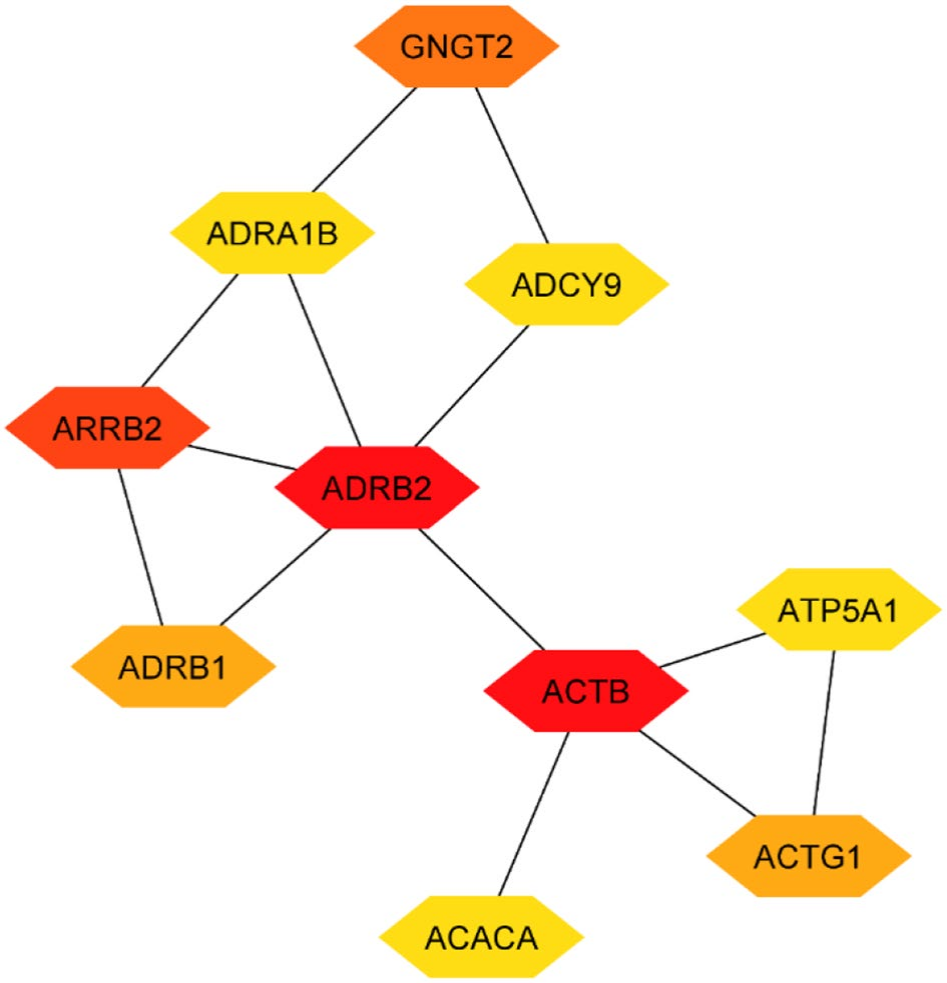
Top 10 hub genes network*.*

Notable top 10 hub genes include ADRB2, ACTB, ARRB2, GNGT2, ADRB1, ACTG1, ACACA, ATP5A1, ADCY9 and ADRA1B. These genes demonstrate strong interconnectivity and exert significant influence on the development and progression of lung cancer (see Fig. 5). Our results confirmed the findings of previous studies^51–54^ and identified new genes that could serve as potential biomarkers for lung cancer. In the network, nodes that are colored in red indicate higher values of MCC or node degree. Nodes with colors ranging between red and yellow represent intermediate values of MCC or node degree, while nodes colored in yellow indicate lower values of MCC or node degree. Table 8 illustrates the configuration of PPI network we used.

**Table 8:** Network analysis configuration.

Summary statistics	
Number of nodes	49
Number of edges	32
Avg. number of neighbors	2.500
Network diameter	7
Network radius	4
Characteristic path length	3.236
Clustering coefficient	0.247
Network density	0.109
Network heterogeneity	0.673
Network centralization	0.213
Connected components	24
Analysis time (sec)	0.151

### WGCNA result

**Figure 6:**
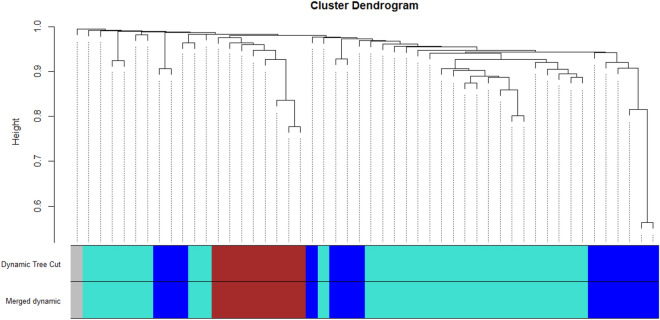
Dendrogram showing the clustering of DEGs using a dissimilarity measure.

In order to explore the clinical relevance of gene modules displaying robust correlations with lung cancer-related clinical attributes, we conducted an in-depth analysis to assess the connections between Module Eigengenes and a range of clinical traits, including class, stage, race, gender, and age. This analysis revealed that four modules exhibited significant associations with the mentioned clinical characteristics, as indicated by the correlation *R*-value. The discovery of crucial modules linked to the onset of lung cancer tumors involved the creation of clustering dendrograms for genes, utilizing topological overlap as a measure of dissimilarity, and assigning distinct module colors. Accordingly, four co-expression modules were established and are visually represented in various colors. Furthermore, the eigengene dendrogram and heatmap were employed to identify sets of correlated eigengenes, referred to as meta-modules (Fig. 6). The findings showed that the four modules could be primarily grouped into two clusters based on their correlations. Consequently, the gray module was identified as the key module and consequently can be selected for further analysis. The module-trait relationship arises from the correlation between modules and various clinical traits such as class, stage, race, gender, and age. The various colors on the left side correspond to distinct modules (MEbrown, MEblue, MEturquoise, and MEgrey). On the right side illustrates a ranking indicating the correlation coefficient. Each column corresponds to a clinical characteristic, and each cell within the matrix displays the corresponding correlation. A negative value in a cell signifies a negative correlation (Fig. 9).

**Figure 7:**
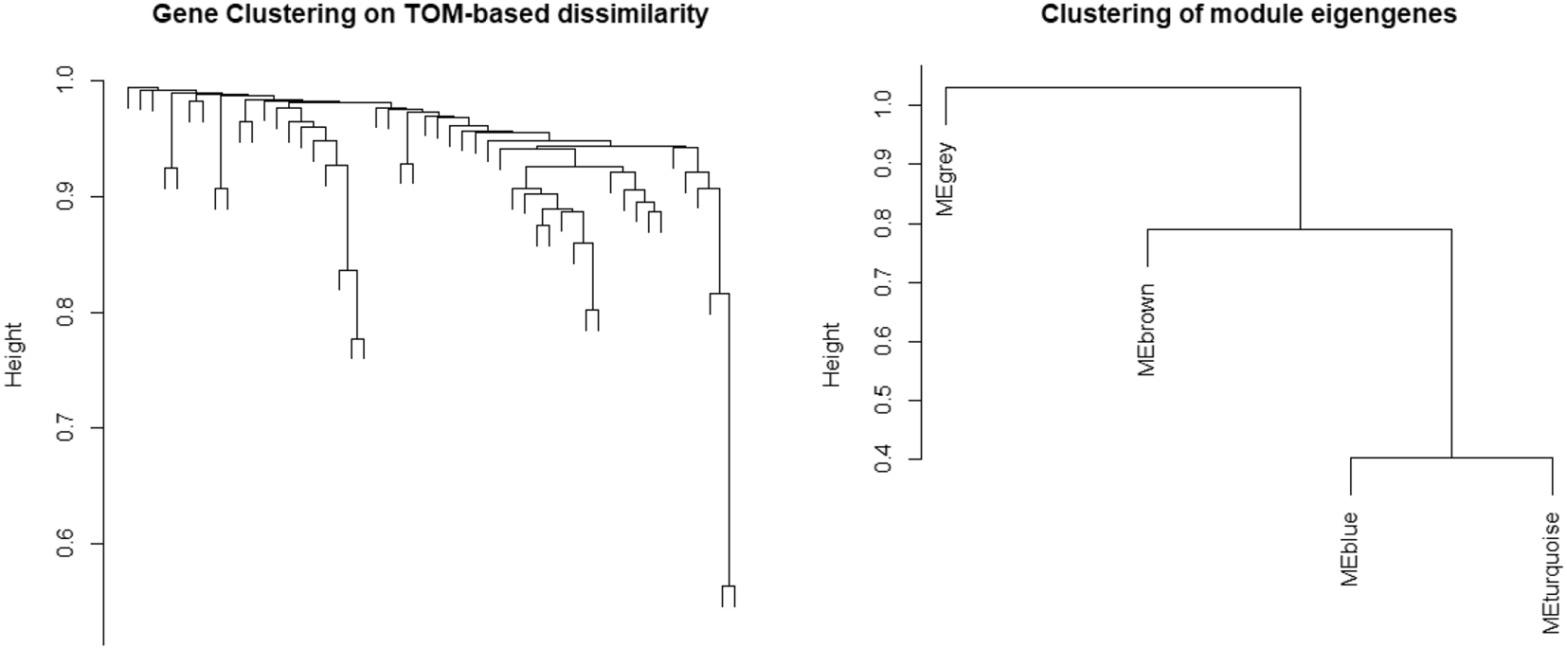
The plot on the left visually displays the clustering of genes through the utilization of dissimilarity measures based on topological overlap (TOM). On the right, the plot illustrates a hierarchical clustering dendrogram, revealing the relationships among module eigengenes. In this representation, nodes are labeled according to their respective module color names, providing insights into the interconnectedness within the eigengene network.

**Figure 8:**
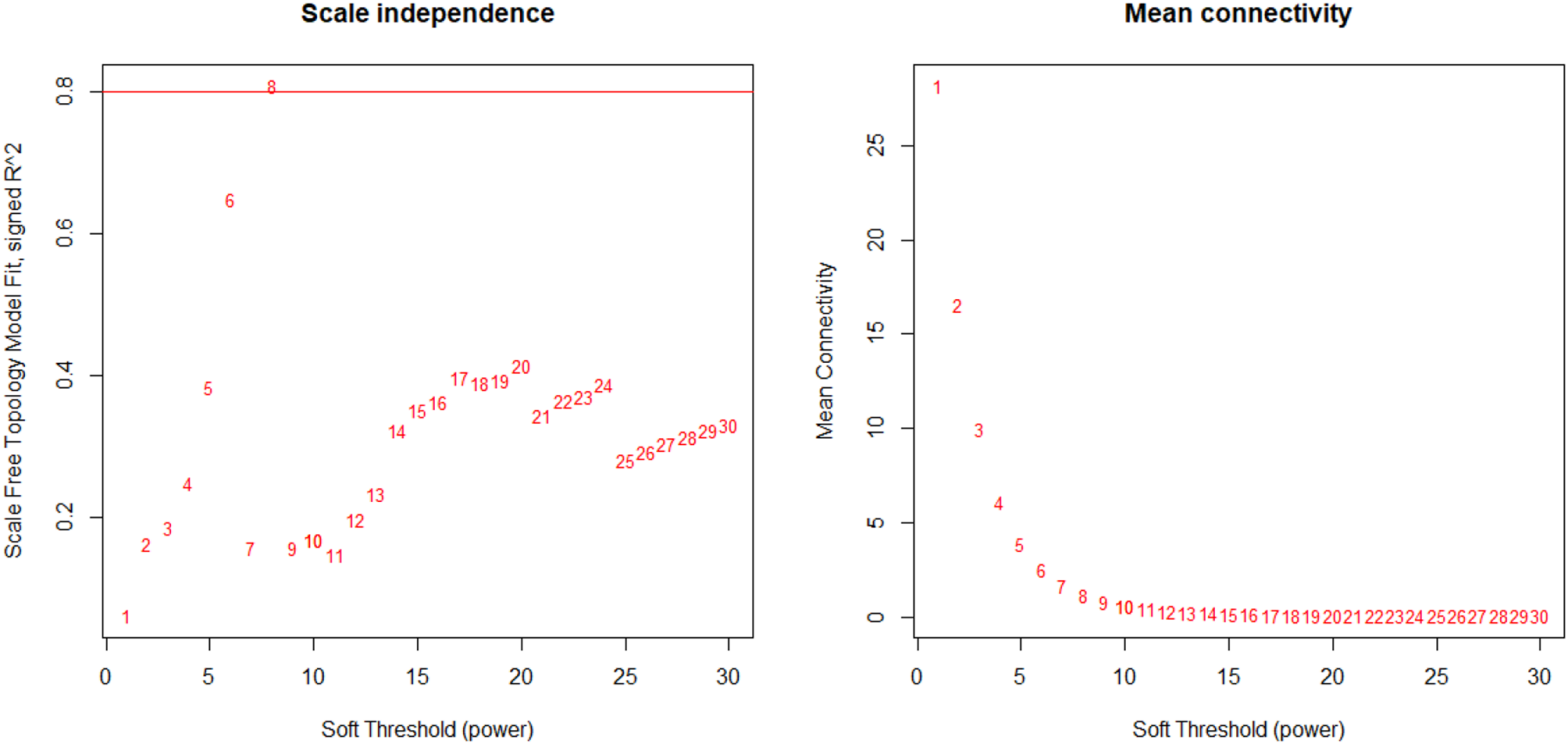
The plot on the left visually presents the outcome of power values in relation to the scale-independence of genes within co-expression modules associated with lung cancer. On the right, the plot showcases the influence of power values on the average connectivity of genes within co-expression modules related to lung cancer.

Figure 7 depicts the gene clustering based on TOM (Topological Overlap Matrix) dissimilarity. The left side of the figure provides a visual representation of gene clustering using dissimilarity measures derived from topological overlap. Meanwhile, the right side displays a hierarchical clustering dendrogram, offering insights into the relationships among module eigengenes. Moving on to Fig. 8, the left plot illustrates the impact of power values on the scale-independence of genes within co-expression modules associated with lung cancer. On the right, the plot demonstrates how power values influence the average connectivity of genes within co-expression modules linked to lung cancer.

**Figure 9:**
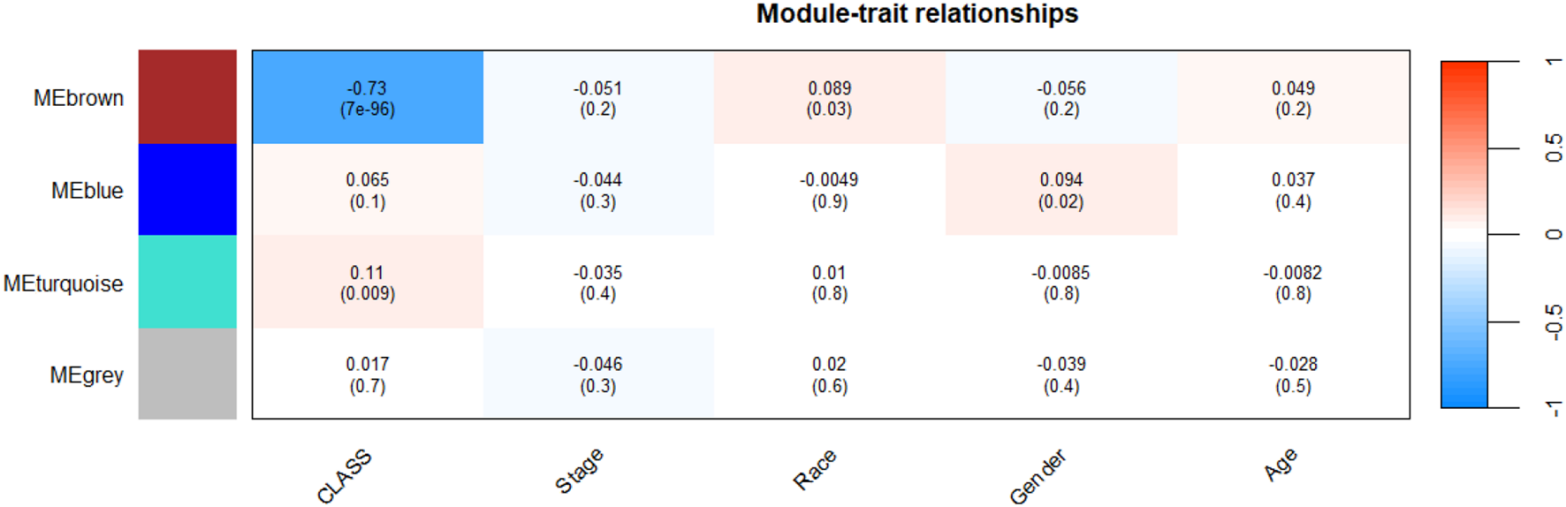
Correlation Heatmap between clinical attributes and module eigengenes based on lung cancer.

Figure 9 illustrates the correlation heatmap that displays the relationships between clinical attributes and module eigengenes in the context of lung cancer. Each cell in the heatmap represents a correlation score along with its corresponding P-value. In the heatmap, red color indicates a positive correlation, while blue color signifies a negative correlation.

### Strength and limitations of the VBEOSA model

The VBEOSA model has strengths in its ability to leverage multiple classification models through a voting mechanism, thus enhancing feature selection accuracy and robustness. However, its limitations include reliance on the quality and diversity of individual models, and the assumption of equal reliability among models. Future work can involve integrating more diverse classification models, addressing imbalanced datasets, exploring applications beyond gene expression data, and refining the voting mechanism through adaptive weights or dynamic adjustments. These efforts aim to improve the VBEOSA model’s performance, generalizability, and applicability in various classification problems.

### Summary of findings

Feature selection plays a crucial role in the analysis of high-dimensional datasets such as gene expression data in lung cancer research. In this study, we proposed a novel ensemble-based approach called the Voting Binary Ebola Optimization Search Algorithm (VBEOSA) to address the challenges of feature selection and classification in lung cancer. The algorithm combines the power of binary optimization and Ebola optimization search and leverages popular classification models, including Support Vector Machines (SVM), Decision Trees (DT), k-Nearest Neighbors (KNN), Random Forest (RF), Multi-Layer Perceptron (MLP), and Gaussian Naïve Bayes (GNB), through a soft voting mechanism to generate robust predictions. We applied VBEOSA to a lung cancer gene expression dataset obtained from the Cancer Genome Atlas (TCGA) repository. Before the feature selection process, we performed preprocessing steps to clean and prepare the dataset. This included outlier detection using array-array intensity correlation, normalization of gene expression data, and filtration based on mean expression values. The selected features were then used to identify hub genes related to lung cancer using protein–protein interaction (PPI) analysis methods.

Through PPI analysis, we identified the top 10 hub genes associated with lung cancer using MCC, including ADRB2, ACTB, ARRB2, GNGT2, ADRB1, ACTG1, ACACA, ATP5A1, ADCY9, and ADRA1B. These hub genes are found to be significantly involved in lung cancer based on enrichment analysis. Pathway analysis reveals the most significant pathways associated with lung cancer, including Salivary secretion, Dilated cardiomyopathy, Renin secretion, Arrhythmogenic right ventricular cardiomyopathy, Vibrio cholerae infection, cGMP-PKG signaling pathway, Regulation of lipolysis in adipocytes, Vascular smooth muscle contraction, Calcium signaling pathway, and Circadian entrainment. Our results demonstrate the effectiveness of VBEOSA in selecting informative features and identifying key hub genes and pathways associated with lung cancer. This study contributes to a better understanding of the molecular mechanisms underlying lung cancer and provides insights into potential diagnostic and therapeutic targets.

## Conclusion

In this study, we harnessed the potential of the VBEOSA algorithm to identify 50 significant genes closely linked to lung cancer. Further exploration through protein–protein interaction (PPI) analysis led to the identification of a select group of 10 hub genes, ADRB2, ACTB, ARRB2, GNGT2, ADRB1, ACTG1, ACACA, ATP5A1, ADCY9, and ADRA1B, each of which plays a pivotal role in the context of lung cancer, as indicated by MCC analysis. Enrichment analysis provided strong confirmation of the substantial involvement of these hub genes in the disease. Notably, our research not only validated prior studies but also unearthed promising novel biomarkers for lung cancer. Pathway analysis shed light on several significant pathways, including Salivary secretion, Dilated cardiomyopathy, Renin secretion, among others, offering insights into the underlying molecular mechanisms of lung cancer. These findings bear significant implications for enhancing the diagnosis, prognosis, and the development of therapeutic strategies for lung cancer. In addition, our use of the WGCNA method revealed a distinct "gray module" displaying a particularly robust association with lung cancer, which was subsequently chosen for in-depth analysis.

## Data Availability

The datasets used and/or analyzed during the current study are available from the corresponding author on reasonable request.

## Abbreviations

AAIC: Array-array intensity correlation
ABC: Artificial bee colony
ACO: Ant colony optimization
ADC: Adenocarcinoma
AMD: Age-related macular degeneration
BEOSA: Binary ebola optimization search algorithm
CNS: Central nervous system dataset
CRC: Colorectal cancer
CS: Cuckoo search
DE: Differential evolution
DEGs: Differentially expressed genes
DLBCL: Dfiuse large B-cell lymphoma
DNA: Deoxyribonucleic acid
DT: Decision trees
ECM: Extracellular matrix
EOSA: Ebola optimization search algorithm
FFA: Firefly algorithm
FS: Feature selection
GA: Genetic algorithms
GEO: Gene expression omnibus
GNB: Gaussian Naïve Bayes
GO: Gene ontology
HFSIA: Hybrid feature selection method based on artificial immune algorithm optimization
ICA: Independent component analysis
JMO-FSCD: Multi-objective optimization for feature selection and classifier design
KEGG: Kyoto encyclopedia of genes and genomes
KNN: K-nearest neighbors
LOOCV: Leave-one-out cross-validation
MCC: Maximal clique centrality
MCN: Maximum neighborhood component
MLP: Multi-layer perceptron
NB: Naive Bayes
NSCLC: Non-small cell lung cancer
NT: Nested transfer
PaCa: Pancreatic cancer
PPI: Protein–protein interaction
PSO: Particle swarm optimization
RF: Random forest
RNA: Ribonucleic acid
SA: Simulated annealing
SCC: Squamous cell carcinoma
SCLC: Small cell lung cancer
SVM: Vector machines
TCGA: The cancer genome Atlas
TS: Tabu search
VBEOSA: Voting-based enhanced binary ebola optimization search algorithm
WGCNA: Weighted gene co-expression network analysis
